# Going beyond Poisson processes: a new statistical framework in neuronal modeling and data analysis

**DOI:** 10.1186/1471-2202-14-S1-P332

**Published:** 2013-07-08

**Authors:** Taşkın Deniz, Stefan Rotter

**Affiliations:** 1 Bernstein Center Freiburg & Department of Biology, Freiburg University, Freiburg, 79104, Germany

## 

Cortical spike trains *in vivo *often show a high irregularity, reflected by a coefficient of variation (CV) close to one [1]. Such irregular single neuron spiking has consequently been associated with Poisson processes, and many models involving neuronal spike trains inherited this ideology. This viewpoint is further supported by the Palm-Khintchine theorem, which states that a superposition of a large number of renewal processes with very small intensity behaves like a Poisson process. It was demonstrated, however, that this theorem doesn't always apply to the superposition of neuronal spike trains [2-4]. Moreover, Poisson processes lack the temporal properties observed in population responses to input modulation either via a stimulus *in vivo *[5] or via electrical stimulation *in vitro *[6].

In this study, we report on new techniques to deal with non-Possonian aspects of stationary neuronal spike trains, as well as non-equilibrium population responses [7], based on Markov point processes (MPP), commonly known as continuous time Markov chains (CTMC). We compute the interspike interval (ISI) distribution by algebraically solving the first passage time problem for MPP neuron models, and compute the transient population responses with a similar technique. The same technique is used to compute exact cross-correlation functions for a shared input paradigm [8]. We advertise MPPs as a new powerful framework in neural network modeling and neural data analysis with many possible applications.
